# *In Vitro* Activity of Rifampicin and Verapamil Combination in Multidrug-Resistant *Mycobacterium tuberculosis*

**DOI:** 10.1371/journal.pone.0116545

**Published:** 2015-02-17

**Authors:** Fernanda de Oliveira Demitto, Renata Claro Ribeiro do Amaral, Flaviane Granero Maltempe, Vera Lúcia Dias Siqueira, Regiane Bertin de Lima Scodro, Mariana Aparecida Lopes, Katiany R. Caleffi-Ferracioli, Pedro Henrique Canezin, Rosilene Fressatti Cardoso

**Affiliations:** 1 Postgraduation in Health Sciences, State University of Maringa, Avenida Colombo, 5790, Maringa, Parana, 87020–900, Brazil; 2 Postgraduation in Bioscience and Pathophysiology, State University of Maringa, Avenida Colombo, 5790, Maringa, Parana, 87020–900, Brazil; 3 Laboratory of Medical Bacteriology, Department of Clinical Analysis and Biomedicine, State University of Maringa, Avenida Colombo, 5790, Maringa, Parana, 87020–900, Brazil; Indian Institute of Science, INDIA

## Abstract

The aim of the present study was to evaluate the effect of the combination of rifampicin (RIF) and verapamil (VP) against the *Mycobacterium tuberculosis* H_37_Rv reference strain and six multidrug-resistant (MDR) *M. tuberculosis* clinical isolates by determining Time-Kill Curves and the ability to efflux drug by fluorometry. The RIF+VP combination showed synergism in one MDR clinical isolate. For the other five MDR clinical isolates, the drug combination showed no interaction. The MDR clinical isolate had lower ethidium bromide (EtBr) accumulation when exposed to the RIF+VP combination, compared with RIF and VP exposure alone. The other MDR clinical isolates showed no significant difference in EtBr accumulation. These results suggest greater efflux action in one of the MDR clinical isolates compared with the *M. tuberculosis* H_37_Rv reference strain. The other five MDR isolates may have additional mechanisms of drug resistance to RIF. The use of the RIF+VP combination made one MDR bacillus more susceptible to RIF probably by inhibiting efflux pumps, and this combination therapy, in some cases, may contribute to a reduction of resistance to RIF in *M. tuberculosis*.

## Introduction

The World Health Organization estimated that 8.6 million people developed tuberculosis (TB) in 2012, and 1.3 million died, maintaining the disease as a major global health problem [1].

One of the problems in combating TB is the intrinsic and acquired resistance of *Mycobacterium tuberculosis* to therapeutic agents, hindering the development of new drugs and therapeutic approaches [2]. Researchers have sought to understand the mechanisms of drug resistance, mainly in multidrug-resistant (MDR) and extensively drug-resistant (XDR) *Mycobacterium tuberculosis*. The former is defined as resistance to at least the two first-line drugs, isoniazid (INH) and rifampicin (RIF). The latter is defined as resistance to INH and RIF, with additional resistance to fluoroquinolones and at least one of the three injectable second-line drugs (kanamycin, amikacin, and capreomycin) [1–3].

Rifampicin is well known to be the backbone of modern anti-TB chemotherapy. Bacilli that are resistant to RIF have become a serious health problem worldwide. Resistance to anti-TB drugs has been attributed to mutational alterations of the drug biotarget [4]. For RIF, mutations in the *rpoB* gene have been shown to cause resistance in 95–98% of RIF-resistant *M. tuberculosis* isolates [5]. However, evidence suggests that efflux systems also play an important role in drug resistance in *M. tuberculosis* [6–8].

Bacterial efflux pumps (EPs) are membrane proteins that are able to actively extrude a broad range of substrates, including drugs, from the cytoplasm to the external bacterial environment and can be inhibited by EP inhibitors (EPIs) [9].

One well-known EPI is verapamil (VP), an inhibitor of MDR P-glycoprotein, which is an adenosine triphosphate (ATP)-binding cassette transporter that influences the cellular accumulation of antiretroviral and anticancer drugs, and its inhibitory activity against mycobacterial EPs has been previously demonstrated [10–11].

The aim of the present study was to evaluate the synergism of a RIF+VP combination by determining time-kill curves and drug efflux activity in the *M. tuberculosis* H_37_Rv reference strain and MDR *M. tuberculosis* clinical isolates.

## Materials and Methods

### Bacterial samples

Six MDR *M. tuberculosis* clinical isolates (18, 19, 64A, 71A, 109, and 3614) were previously genotypically differentiated according to Mycobacterial Interspersed Repetitive Units (MIRU) [12] and spoligotyping [13] (Table 1). The RIF+VP combination was shown to have a synergic effect (fractional inhibitory concentration: 0.25–0.37) in a modified checkerboard assay (i.e., the Resazurin Drugs Combination Microtiter Assay [REDCA]) [14]. All of the studied MDR clinical isolates belong to the reference center for TB diagnosis (Laboratory of Teaching and Research in Clinical Analysis, State University of Maringa, Parana, Brazil), and drug resistance to RIF was determined by the Lowenstein-Jensen proportion method [15]. The wildtype reference strain *M. tuberculosis* H_37_Rv (ATCC 27294) was used as a control.

**Table 1:** Molecular characterization, drug susceptibility profile, minimum inhibitory concentration, and drug interaction in the *M. tuberculosis* H_37_Rv reference strain and multidrug-resistant clinical isolates.

**Clinical isolate**	**MIC RIF** **(μg/ml)**	**MIC VP** **(μg/ml)**	**MIRU-VNTR**	**Spoligotyping**	**Drug susceptibility profile**	**MIC EtBr (μg/ml)**	**REDCA** **FICI** **RIF/VP**
H_37_Rv	0.004	125	NP	777777477760771	Susceptible	1	0.75
64A	25	125	124325163322	677737607760771	(INH, RIF)^R^	0.5	**0.37**
71A	50	125	225313153323	777777777720771	(INH, RIF, PZA)^R^	1	**0.25**
19	25	62.5	224 327153324	776177607760771	(INH, RIF, EMB)^R^	0.5	**0.37**
18	50	125	224326153324	776177607760771	(INH, RIF, EMB)^R^	0.5	**0.25**
109	25	125	224326153325	776177607760771	(INH, RIF)^R^	0.5	**0.37**
3614	12.5	62.5	224225163321	677737607760771	(INH, RIF, EMB)^R^	0.5	**0.37**

R, resistant; NP, not performed; INH, isoniazid; RIF, rifampicin; EMB, ethambutol; PZA, pirazinamid; FICI, fractional inhibitory concentration index; REDCA; Resazurin Drugs Combination Microtiter Assay. VP, verapamil; EtBr, ethidium bromide. Numbers in bold represent synergism.

### Antimicrobial and efflux pump inhibitor agents

Rifampicin (Sigma, St. Louis, MO, USA) and VP (Sigma, St. Louis, MO, USA) stock solutions were freshly prepared at concentrations of 2,000 and 20,000 μg/ml, respectively. Verapamil was prepared in distilled water, and RIF was prepared in methanol:water (1:10, v/v). The drug solutions were sterilized by filtration through 0.22 μm filters (Millipore, Billerica, MA, USA). Additional dilutions were performed in Middlebrook 7H9 medium (Difco Laboratories, Detroit, MI, USA) supplemented with oleic acid, bovine serum albumin, dextrose, and catalase (OADC) enrichment (BBL/Becton-Dickinson, Sparks, MD, USA) to reach RIF concentrations of 0.002–500 μg/ml and VP concentrations of 15.62–1,000 μg/ml. The RIF and VP concentrations were determined according to the minimum inhibitory concentrations (MICs) for the *M. tuberculosis* H_37_Rv reference strain and MDR clinical isolates that were previously determined by the Resazurin Microtiter Plate Assay (REMA) [16].

### Time-kill studies

The MDR clinical isolates and *M. tuberculosis* H_37_Rv reference strain were first grown in Middlebrook 7H9 medium (Difco Laboratories, Detroit, MI, USA) supplemented with oleic acid, bovine serum albumin, dextrose, and catalase (OADC) enrichment (BBL/Becton-Dickinson, Sparks, MD, USA) with 0.2% glycerol (v/v) and 0.025% Tween 80 (v/v) to 1 McFarland standard turbidity (3 × 10^8^ colony-forming units [CFU]/ml) for 15 days at 35–37°C. The cell suspensions were then adjusted to a final concentration of 10^6^ CFU/ml in OADC-supplemented Middlebrook 7H9 medium. Individual RIF and VP and RIF+VP combination drug solutions were added to each mycobacterial suspension to achieve 0.5 × MIC of the drugs.

The cultures were incubated at 35–37°C with shaking at 96 rotations per minute (rpm) for 7 days. Aliquots (0.1 ml) were removed on the initial day of the experiment and then on the first, third, fifth, and seventh days of incubation and serially diluted (10^-1^, 10^-3^, and 10^-5^) in OADC-supplemented Middlebrook 7H9 medium to avoid drug carry-over. An aliquot (20 μl) of each dilution was seeded on OADC-supplemented Middlebrook 7H11. The plates were incubated at 35–37°C for 21 days, and the colonies were counted. Time-Kill Curve assays were performed three times on different days. The results are expressed as the mean of the three assays. Synergism was defined as a decrease of 2 or more log_10_ CFU/ml compared with the single agent. A decrease in CFU/ml between 2 log_10_ and 1 log_10_ was indicative of an additive interaction [17, 18].

### Efflux assay

Ethidium bromide (EtBr) accumulation was assessed by fluorometry for the MDR *M. tuberculosis* isolates and H_37_Rv reference strain [19, 20]. MDR *M. tuberculosis* isolates and the H_37_Rv reference strain were grown in OADC-supplemented Middlebrook 7H9 medium at 35–37°C until an optical density at 600 nm (OD_600_) of 0.6–0.8 was reached. The cultures were exposed to the 0.5 × MIC of VP, RIF, and RIF+VP that was previously determined by REMA [16] (Table 1) and incubated at 35–37°C for 7 days. Aliquots (900 μl) on the initial, first, third, fifth, and seventh days of incubation were removed and centrifuged at 12,880 × *g* for 3 min. The pellet was rinsed in phosphate-buffered saline (PBS; pH 7.4) with 0.05% Tween 80 (Synth, Diadema, SP, Brazil), and the OD_600_ was adjusted to 0.4 with PBS. Aliquots (100 μl) of the bacillus suspension were transferred to a 96-well plate that contained 0.25 μg/ml EtBr (0.5 × MIC; Sigma-Aldrich Química SA; Table 1). Fluorescence was determined for the bacterial suspension in the absence of drug as a reference assay. Fluorescence relative to EtBr-loaded cells was acquired every 51 s for 60 min at 35–37°C for each drug exposure time using a VICTOR^2^ D fluorometer (PerkinElmer, Santa Clara, CA, USA). The excitation wavelengths were 530/25 nm, and the detection wavelengths were 590/20 nm [18]. The relative fluorescence values were obtained by normalizing the data against the background fluorescence of EtBr [21, 22].

## Results

**Fig 1:**
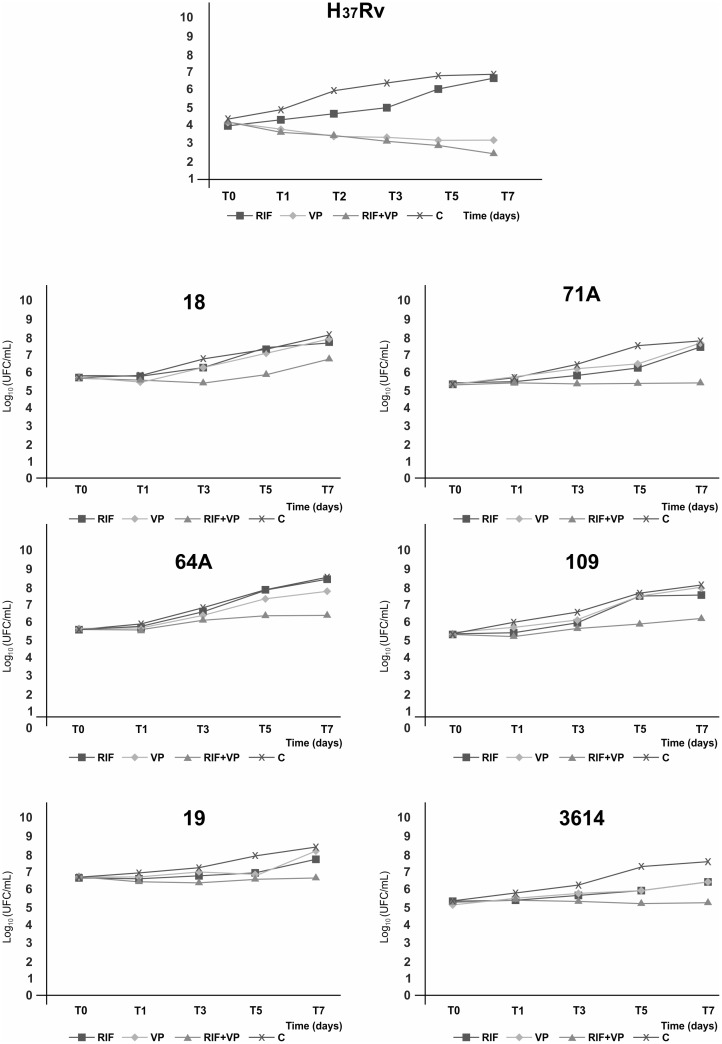
Time-kill curve of the *Mycobacterium tuberculosis* H_37_Rv reference strain and multidrug-resistant clinical isolates 71A, 18, 19, 109, 3614, and 64A exposed to rifampicin (RIF), verapamil (VP), and RIF+VP combination for 7 days at 35–37°C.

In contrast to the REDCA performed previously, a synergistic effect of the RIF+VP combination was observed for only one of the MDR *M. tuberculosis* isolates (71A) in the time-kill curve assay, reflected by a decrease of more than two log_10_ CFU/ml on the seventh day of exposure to the drug combination. For the other five MDR clinical isolates and *M. tuberculosis* H_37_Rv reference strain, the effect of the drug combination did not show an interaction (Fig. 1).

**Fig 2:**
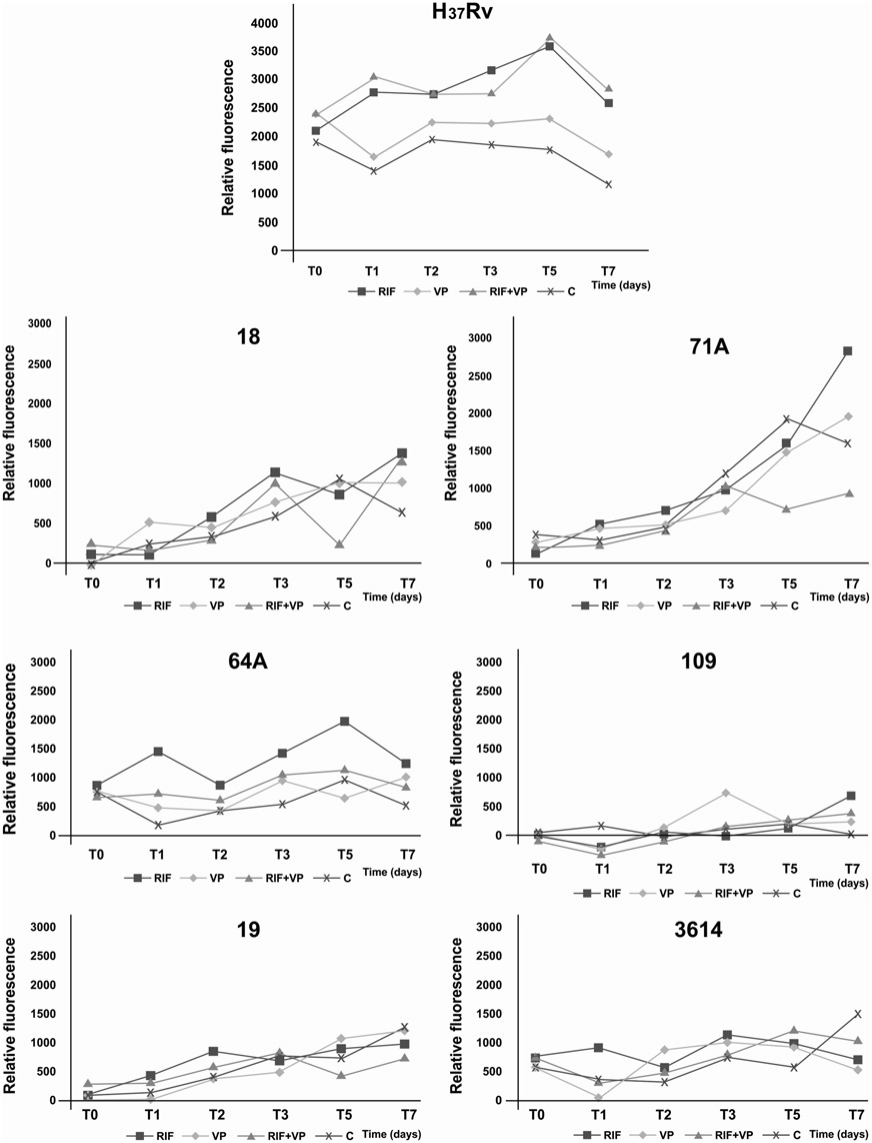
Fluorometry assay. Accumulation of EtBr in the *Mycobacterium tuberculosis* H_37_Rv reference strain and multidrug-resistant clinical isolates 71A, 18, 19, 109, 3614, and 64A. The mycobacteria were loaded with 0.25 μg/ml EtBr in the presence of 0.5 × MIC of verapamil (VP), rifampicin (RIF), and RIF+VP combination for 7 days at 35–37°C.

The results of the EtBr efflux analysis of the *M. tuberculosis* H_37_Rv reference strain and MDR clinical isolates on the initial, first, third, fifth, and seventh days of incubation with VP, RIF, and the RIF+VP combination are shown in Fig. 2. The MDR *M. tuberculosis* clinical isolate 71A exhibited a different EtBr accumulation profile during the incubation time compared with the other five MDR isolates and *M. tuberculosis* H_37_Rv reference strain.

## Discussion

Mutations in drug resistance-associated genes can partially explain the molecular development of drug resistance to some drugs in *M. tuberculosis*. For RIF, specific mutations in the *rpoB* gene that encodes the β subunit of DNA-dependent RNA polymerase, which is the main biotarget of RIF, may result in resistant bacilli. Approximately 2–5% of RIF-resistant *M. tuberculosis* isolates do not harbor mutations in this target gene. Therefore, the modulatory actions of drug EPs have been shown to be relevant in the resistance to RIF in this bacillus.

The present study evaluated the synergistic effects of the RIF+VP combination by determining time-kill curves and the activity of EPs in MDR *M. tuberculosis* clinical isolates, which previously showed synergism based on the checkerboard method. Time-kill curves reflect the bactericidal effect of a drug and can be used to evaluate synergism between two or more drugs [20], and the fluorometry assay evaluates the inhibitory action of EPIs on EPs based on the accumulation of EtBr, a fluorescent substrate, inside the cell. When inside the cell, EtBr can bind to numerous targets, and the balance between influx and efflux can be estimated in real time in the presence or absence of EPIs [22].

In the present study, the RIF+VP combination did not improve the activity of RIF in the susceptible *M. tuberculosis* H_37_Rv reference strain. In this strain, RIF exhibited time-dependent killing activity, which was clearly observed after the fifth day of RIF exposure and consistent with previous studies [23]. The time-kill curve assays of the six MDR isolates revealed the growth of bacilli after the fifth day of exposure to RIF alone or the RIF+VP combination, which was not observed with the susceptible *M. tuberculosis* H_37_Rv reference strain. The reason why MDR isolates begin to grow after that exposure time is still unclear, but the EtBr accumulation assay suggests the involvement of EPs.

Variations in the EtBr accumulation profile were observed throughout the incubation time among the MDR *M. tuberculosis* clinical isolates, demonstrated by the fluorometry assay. The *M. tuberculosis* H_37_Rv reference strain, which is a well-known susceptible reference strain, had higher EtBr accumulation compared with the MDR *M. tuberculosis* isolates. This result led us to infer the presence of EP actions in the MDR isolates, in which the addition of the RIF+VP combination increased EtBr accumulation. In general, for all of the MDR isolates, EtBr accumulation was lower and occurred time-independently compared with the susceptible strain (H_37_Rv).

For the MDR isolate 71A, the RIF+VP combination had a synergistic effect, indicated by the time-kill curve assay, and a very different EtBr accumulation profile was observed after the third day of drug exposure. The fluorometry assay showed that the exposure of this MDR isolate to VP alone caused high EtBr accumulation. This effect may be attributable to the blockade of EP action and interference with the efflux system. In the cultures that were exposed to the RIF+VP combination, lower EtBr accumulation was observed with the same incubation time. Lower EtBr accumulation may be attributable to fewer EPs that were still available to the action of VP because some EPs were performing RIF extrusion. Another possibility is that the expression of EPs may be increased by the stress caused by RIF exposure. The present findings are consistent with Jiang [7], in which the ABC superfamily ATP-binding cassette EP, encoded by the *pstB* gene, was overexpressed in the presence of RIF in *M. tuberculosis*.

The present results corroborate previous studies [24–26], in which the constitutive or inducible expression of efflux systems in response to treatment with RIF may contribute to a decrease in the intracellular concentration of RIF and consequently the development of *M. tuberculosis* drug resistance. Additionally, the efflux-mediated response may provide an early stress response that creates an opportunity for other resistance mechanisms to arise [27].

The contributory role of EPs in drug resistance in *M. tuberculosis* may be extended to other anti-TB drugs, including combinations of INH and other EPIs (e.g., INH and EMB) [27, 28] and ofloxacin+VP [28–31], which showed promising results in killing *M. tuberculosis in vitro*. Sharma et al. [8] studied a combination of RIF and piperine (i.e., an EPI) and reported a reduction of the MIC values of RIF in the *M. tuberculosis* H_37_Rv reference strain and MDR clinical isolates. The results obtained by these authors corroborate a previous study by Piddock et al. [32], who observed an increase in the intracellular concentration of RIF in *M. tuberculosis* and other mycobacteria using a RIF+reserpine combination, suggesting a role for EPIs in enhancing the activity of RIF in some species of mycobacteria. However, to our knowledge, no *in vitro* study with a RIF+VP combination has been performed in MDR *M. tuberculosis* clinical isolates.

Studies of nontuberculous mycobacteria (NTM) have shown a significant impact of EPIs in reducing the resistance to drugs that are used to treat this kind of infection. Rodrigues et al. [33] performed an *in vitro* study using a microdilution method and fluorometry and found a significant reduction of resistance to clarithromycin and erythromycin in *M. avium* ATCC 25291 in the presence of VP. Jin et al. [34] found that the EPIs farnesol, carbonyl cyanide-*m*-chlorophenyl-hydrazone, reserpine, chlorpromazine, and VP caused significant EtBr accumulation in *M. smegmatis*, and the most pronounced effect was induced by VP.

The *in vitro* findings of the present study corroborate previous studies that evaluated anti-TB drug and EPI combinations *in vivo*, showing promising results in the treatment of TB. Louw et al. [35] observed a significant reduction of lung bacillus loads in BALB/c mice that were infected with an MDR strain after 1–2 months of treatment using the combination of VP and first-line anti-TB drugs (RIF, INH, and pyrazinamide). Additionally, Gupta et al. [36] evaluated the pharmacokinetic interactions between RIF and VP in mice infected with *M. tuberculosis* H_37_Rv and concluded that standard TB chemotherapy combined with VP accelerated bacillus clearance, with near sterilization and significantly lower relapse rates in just 4 months.

Finally, coadjutant therapy with VP inhibited mycobacterial EPs. This renders the bacillus more susceptible to RIF and may reduce the probability of the selection of spontaneously arising mutants. Additional studies are required to elucidate the mechanism of action of the RIF+VP combination and evaluate the safety and efficacy of the combination for patients infected with MDR *M. tuberculosis*.
