# Correction: *In Vitro* Activity of Rifampicin and Verapamil Combination in Multidrug-Resistant *Mycobacterium tuberculosis*

**DOI:** 10.1371/journal.pone.0133343

**Published:** 2015-07-24

**Authors:** Fernanda de Oliveira Demitto, Renata Claro Ribeiro do Amaral, Flaviane Granero Maltempe, Vera Lúcia Dias Siqueira, Regiane Bertin de Lima Scodro, Mariana Aparecida Lopes, Katiany R. Caleffi-Ferracioli, Pedro Henrique Canezin, Rosilene Fressatti Cardoso

The graph legends for Figs 1 and 2 are incorrect. The symbols for RIF and VP are switched. Please see the complete, correct Figs 1 and 2 here.

**Fig 1:**
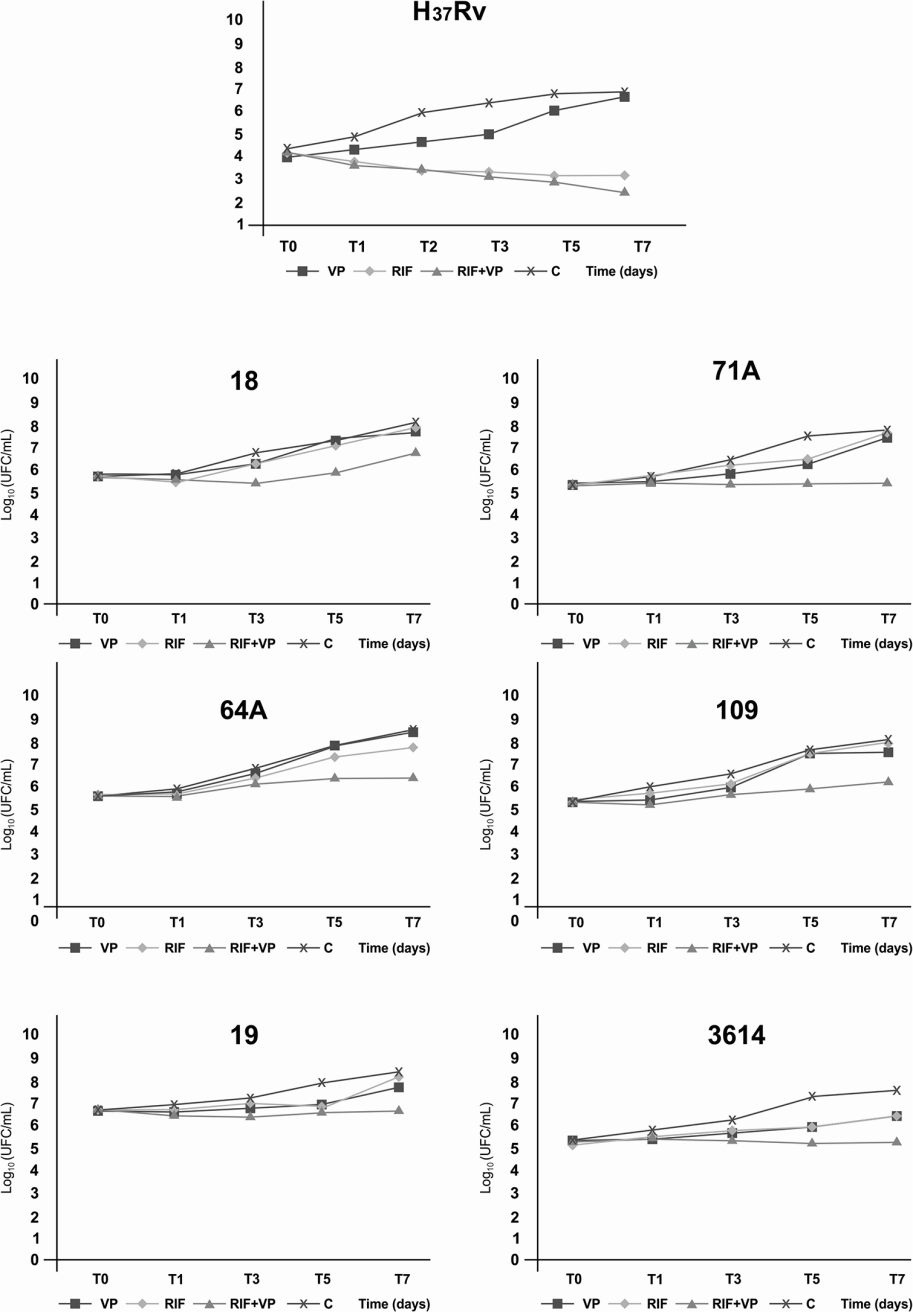
Time-kill curve of the *Mycobacterium tuberculosis* H_37_Rv reference strain and multidrug-resistant clinical isolates 71A, 18, 19, 109, 3614, and 64A exposed to rifampicin (RIF), verapamil (VP), and RIF+VP combination for 7 days at 35–37°C.

**Fig 2:**
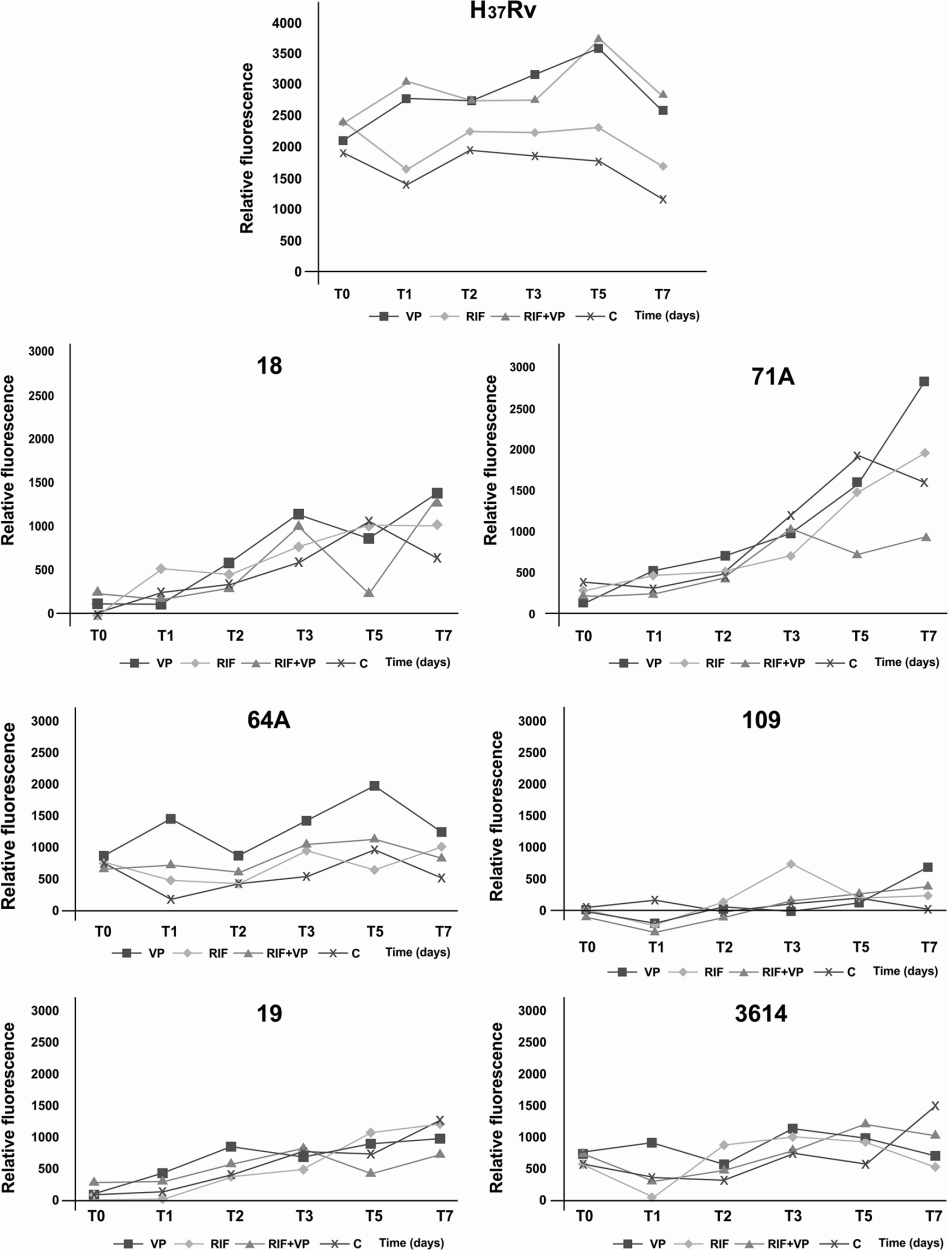
Fluorometry assay. Accumulation of EtBr in the *Mycobacterium tuberculosis* H_**37**_Rv reference strain and multidrug-resistant clinical isolates 71A, 18, 19, 109, 3614, and 64A. The mycobacteria were loaded with 0.25 μg/ml EtBr in the presence of 0.5 × MIC of verapamil (VP), rifampicin (RIF), and RIF+VP combination for 7 days at 35–37°C.
